# Tart Cherry (*Prunus cerasus* L.) Pit Extracts Protect Human Skin Cells against Oxidative Stress: Unlocking Sustainable Uses for Food Industry Byproducts

**DOI:** 10.3390/foods12203748

**Published:** 2023-10-12

**Authors:** Hannah Decot, Meenakshi Sudhakaran, Emma Boismier, Anthony Schilmiller, Ethan Claucherty, Andrea I. Doseff, Bahar Aliakbarian

**Affiliations:** 1 Molecular, Cellular, and Integrative Physiology Graduate Program, Michigan State University, 567 Wilson Rd., East Lansing, MI 48824, USA; decothan@msu.edu (H.D.); sudhaka7@msu.edu (M.S.); 2 Department of Physiology, Michigan State University, 567 Wilson Rd., East Lansing, MI 48824, USA; boismie4@msu.edu; 3 Mass Spectrometry and Metabolomics Core, Michigan State University, 603 Wilson Rd., East Lansing, MI 48824, USA; schilmil@msu.edu; 4 The Axia Institute, Michigan State University, 1910 W. St. Andrews Rd., Midland, MI 49640, USA; clauche9@msu.edu; 5 Department of Pharmacology and Toxicology, Michigan State University, 1355 Bogue St., East Lasing, MI 48824, USA; 6 Department of Biosystems and Agricultural Engineering, Michigan State University, 524 S Shaw Lane, East Lansing, MI 48824, USA

**Keywords:** cell death and survival, food byproduct, flavonoids, plant compounds for health, cosmeceuticals, metabolomics

## Abstract

Industrial processing of tart cherries (*Prunus cerasus* L.) produces bioproducts like cherry pits (CP), which contribute to adverse environmental effects. To identify sustainable strategies to minimize the environmental impact of cherry processing, we investigated their potential value as antioxidants for prospective utilization within cosmeceutical applications. Untargeted metabolomic analyses of water and water: ethanol CP extracts using an eco-friendly technique revealed significant enrichment in coumaroyl derivatives and flavonoids with congruent metabolite representation regardless of the extraction solvent. The antioxidant activity of tart CP extracts was evaluated on human skin cells exposed to H_2_O_2_ or LPS, modeling environmentally induced oxidants. Notably, both CP extracts provide antioxidant activity by reducing H_2_O_2_ or LPS-induced ROS in human skin keratinocytes without affecting cell viability. The CP extracts increased the expression of *CAT* and *SOD1* genes encoding antioxidant regulatory enzymes while decreasing the expression of *NOS2*, a pro-oxidant regulator. These findings reveal the antioxidant properties of tart CP, offering new opportunities to produce natural-based skin care products and adding economic value while providing sustainable options to reduce the environmental impact of food byproducts.

## 1. Introduction

The cherry processing food industry generates a significant amount of waste, including cherry pits, which are often burned or discarded in landfills. According to the United States Department of Agriculture’s National Agricultural Statistics Services (USDA’s NASS) report, 275,000 tons of sweet cherry (*Prunus avium* L.) and 229.2 million pounds of tart cherry (*Prunus cerasus* L.) were produced in the United States in 2022 [1]. Michigan produces about 70% of the total United States tart cherry crop (159.5 million pounds) [2]. More than 90% of tart cherries are processed to produce juice, wine, and brined products [3], resulting in the generation of byproducts such as cherry pomace (15–28% of the initial fruit) [4] and the cherry pit (7–15% of the whole fruit) [5] depending on the process.

Tart cherry pits (CP) contribute to environmental soil and water pollution and greenhouse gas emissions [6]. Thus, solutions to minimize the environmental impact of tart CP waste are greatly needed. Attempts to reduce the environmental impact included the use of CP oil for cooking and cosmetics [7]. CP products have been pyrolyzed to produce biochars used as soil amendments and active biochars to remove metals from drinking water [8]. Other studies showed optimization procedures to extract and disintegrate sour CP insoluble fiber and transform it into soluble dietary fibers [9]. Based on the rapidly expanding cherry production and processing rates, the identification of valuable applications for CP is an environmental and economic necessity. 

The skin protects against biological (e.g., pathogens) and nonbiological (ultraviolet light [UV] or pollutants) agents. Exacerbated exposure to these agents triggers inflammation and reactive oxygen species (ROS) overproduction, inducing cellular damage [10]. For example, exposure to UVB irradiation of keratinocytes leads to the intracellular generation of hydrogen peroxide (H_2_O_2_) [11], which stimulates ROS and inflammatory cytokine production. ROS overproduction has been associated with an increased incidence of dermatological conditions, such as skin cancer, psoriasis, and early aging [12,13,14,15,16]. Therefore, the identification of safe and sustainable approaches to decrease ROS levels to maintain skin homeostasis has attracted great interest. Additionally, in recent years, there has been a growing interest from consumers in natural-based products for skin care compared to synthetic pharmaceuticals [17].

Polyphenols are one of the largest classes of naturally occurring plant metabolites possessing several health benefits [18,19]. Studies have shown that polyphenols exert antioxidant activity against UV-induced H_2_O_2_ and pathogen-derived stimulants such as lipopolysaccharide (LPS), thereby reducing inflammation [18,20,21,22]. Previous studies have shown that tart cherries exhibit antioxidant capabilities due to their high content of polyphenols compared to other varieties of cherries and fruits [23]. Other studies have focused on the benefits of bioactive components from different parts of the cherry, including the stem [17] and seed [24]. However, the antioxidant activity of tart CP for their potential value as antioxidants for prospective utilization within cosmeceutical applications has not been investigated previously. 

Considering the need for natural-based products, the urge to reduce the environmental impacts of food processes, and the lack of a comprehensive study on tart CP valorization, the goal of this work was to assess the potential application of antioxidants extracted from the CP for cosmetic purposes. Human keratinocyte HaCaT cells were used to evaluate the cytotoxicity and anti-inflammatory properties of the most abundant phenolic components found in the extracts, as well as the full CP extracts. By opening a new avenue to produce natural-based skin care products, this study can simultaneously mitigate the environmental impact of cherry processing plants.

## 2. Materials and Methods

### 2.1. Materials and Reagents

Tart cherry (*Prunus cerasus* L.) pits of Montgomery cultivar were kindly provided by a cherry producer and processor (Peterson Farms) located in Shelby, MI, USA. Pits were oven-dried for 24 h at 60 °C and ground using a laboratory mixer. Fine powders were sealed in plastic bags and stored in the refrigerator until analysis. Folin–Ciocalteu reagent, sodium carbonate, ethanol, 2,2-diphenyl-1-picryl-hydrazyl-hydrate (DPPH), 2,2′-azinobis-(3-ethylbenzothiazoline-6-sulphonic acid) (ABTS), Trolox, aluminum chloride, sodium hydroxide, dimethyl sulfoxide (DMSO), and all standard reagents were purchased from Sigma-Aldrich, Saint Louis, MO, USA.

### 2.2. Extraction Process and Colorimetric Characterization of the Extracts

To extract antioxidants from the CP, two different solvents were used, namely 100% water or water: ethanol (1:1 [*v*/*v*]). Extractions were performed at 150 °C for 30 min using a bench-top stirred reactor (Model 4520, Parr Instrument Company, Moline, IL, USA) and a solid-to-liquid ratio of 1:10 (*w*/*v*) following our previous optimization study [25]. Briefly, 30 g of dried pit powder was mixed with 300 mL of extraction solvent and placed in the stirred reactor. The extracts obtained using 100% water or water: ethanol (1:1 [*v*/*v*]) are referred to as cherry pit water (CPW) or cherry pit ethanol (CPE), respectively. Extraction batches were repeated four times. 

After the extraction, samples were separated using a centrifuge (Thermo Scientific Sorvall Legend Centrifuge, Fisher Scientific, Waltham, MA, USA) for 10 min at 12,000 rpm. The supernatants were separated for analysis. Total Polyphenol (TP), Total Flavonoids (TF), and Antiradical Power (ARP) of the extracts were measured using a double-beam Lambda 365 UV–Vis spectrophotometer (PerkinElmer, Shelton, CT, USA). The quantification of TP in the extracts was carried out using the colorimetric Folin–Ciocalteu assay, as previously described [26]. The UV–Vis spectroscopy was employed with a wavelength of 725 nm using a calibration curve utilizing caffeic acid. TP was expressed as milligrams of Caffeic Acid Equivalent (CAE) per gram of Dried Pit (DP) (mg_CAE_/g_DP_) following common practice in food byproducts [27,28]. TF was measured using the aluminum chloride colorimetric assay [29] employing the same UV–Vis above set at 510 nm. A calibration curve was generated using a standard quercetin solution. Quercetin has been widely used as a standard component to represent flavonoids in different fruit extracts, including cherries, cherry pits, bilberries, blueberries, blackberries, and plums [30,31,32]. TF was shown as milligrams of Quercetin Equivalent (QE) per gram of Dried Pit (DB) (mg_QE_/g_DP_). DPPH assay was used to determine the Antiradical Power (ARP) of the extracts [33]. Both the original DPPH solution and extract sample absorbance were recorded at a wavelength of 515 nm using the aforementioned UV–Vis spectrophotometer. The ARP, expressed as mg_DPPH_/mL_Extract_, is equal to the reciprocal of the effective concentration EC50 (1/EC50) at which the initial extract can reduce 50% of the DPPH in solution [25]. Applying a second assay, the antioxidant activity of the extracts against ABTS was determined using a colorimetric method (absorbance 734 nm), as previously described [34] and the results were expressed as mg_Trolox Equivalent_/mL_Extract_. All the analyses using the extracts were repeated three times.

### 2.3. Preparation of Cherry Pit Extracts for Metabolomic Analyses and In Vitro Tests

For all metabolomic and cell-based studies, 5 g of CPE or CPW were freeze-dried in a Freezone bulk tray dryer (Labconco, Kansas City, MO, USA) for 30 h and reconstituted in 4 mL DMSO. Freeze-drying resulted in a dry weight of 600 mg CP extracts.

### 2.4. Metabolomic Analyses of CP Extracts

CPW and CPE extracts were analyzed via liquid chromatography–mass spectrometry (LC-MS/MS). Data were acquired during each run with both fragmenting and non-fragmenting conditions using a Waters Xevo G2-XS Quadrupole–Time of Flight mass spectrometer interfaced with a Waters Acquity UPLC to profile metabolites and quantify phenolic compounds that we could annotate as previously reported [35]. Briefly, 5 µL of the sample was injected onto a Waters Acquity BEH-C18 UPLC column (2.1 × 100 mm), and compounds were separated using the following 20 min gradient: initial conditions were 99% mobile phase A (10 mM ammonium formate in water, pH 2.8) and 1% mobile phase B (acetonitrile) and held for 0.5 min; ramp to 50% B at 12 min; ramp to 99% B at 16 min and hold at 99% B until 18 min; return to 99% A at 18.01 min and hold at 99% A until 20 min. The column was held at 40 °C, and the flow rate was 0.3 mL/min. Flow from the column was split and directed either into the mass spectrometer for MS/MS analysis or into a diode array detector acquiring data between 220–800 nm. Compounds were ionized using electrospray ionization operating in negative ion mode with a capillary voltage of 2 kV, source temp of 100 °C, dissolution temp of 350 °C, and dissolution and cone gas flow rates of 600 and 40 L/h, respectively. Mass spectra were acquired over a range of *m*/*z* 50–2000 using an MS^E^ method with separate acquisition functions for scans (0.2 s/scan) with no collision energy and scans (0.2 s/scan) with a collision energy ramp of 20–80 V. Lockmass correction was performed using leucine enkephalin as the reference compound. External standard curves for absolute quantification were made with the following pure standards: trans-3-*O*-caffeoylquinic acid (chlorogenic acid), caffeic acid, ferulic acid, quercetin, amygdalin, catechin, naringenin, and apigenin. *p*-Coumarylquinate concentrations were estimated using the caffeoylquinate standard curve. Untargeted analysis of the MS data was performed using Progenesis QI software (version 3.0) with the EZinfo statistics package add-on. 

### 2.5. UV–Visible Absorption Spectra

The UV–Visible absorption spectra of the freeze-dried CP extracts, chlorogenic acid, quercetin, and apigenin were recorded using 1.0 cm quartz cells in BioMate 3S UV–Visible spectrophotometer (Thermo Fisher Scientific, Waltham, MA, USA) at a resolution of 1 nm and a scan speed of 200 nm/min within wavelengths 200 to 800 nm, a range used for quantifying polyphenols including flavonoids [36,37]. DMSO was used as the reference solution. Obtaining the complete UV spectra allows for a comprehensive visualization of the entire chemical composition of each extract.

### 2.6. Cell Lines, Cultures, and Treatments

Human keratinocyte (HaCaT) and embryonic mouse fibroblast (NIH3T3) cells were obtained from the American Type Culture Collection (Rockville, MD, USA). Cells were cultured in complete Dulbecco’s Modified Eagle Medium (DMEM; Gibco, Waltham, MA, USA) supplemented with 5% fetal bovine serum (FBS), 100 μg/mL streptomycin, and 100 U/mL penicillin at 37 °C and 5% CO_2_. For viability assays, cells were treated with varying concentrations of CP extracts, pure polyphenols including amygdalin, chlorogenic acid, caffeic acid, catechin, apigenin or quercetin, H_2_O_2_ (Sigma-Aldrich), or LPS (Escherichia coli O111:B4, Sigma-Aldrich) for 24 or 48 h. All pure polyphenols were purchased from Sigma-Aldrich and resuspended in DMSO. For ROS detection and RT-PCR studies, cells were pre-treated with CP extracts, N-acetyl cysteine (NAC, Sigma-Aldrich), or diluent for 3 h prior to the addition of H_2_O_2_ or LPS for 24 h. 

### 2.7. Cell Viability Assays

HaCaT and NIH3T3 cells were seeded at a density of 1 × 10^5^ and 2 × 10^4^ cells/mL, respectively, into a 96-well plate. Cell viability was evaluated using the CellTiter 96 Aqueous One Solution Cell Proliferation Assay (Promega, Madison, WI, USA) following the manufacturer’s instructions as we previously described [38]. The absorbance at 490 nm was evaluated using the BioTek SynergyTM Neo2 Multi-Mode plate reader (Santa Clarita, CA, USA). The percentage of cell viability was calculated as (A_treatment_ − A_blank_)/(A_control_ − A_blank_) × 100%, where A is the absorbance at 490 nm. 

### 2.8. Assessment of Intracellular ROS

HaCaT cells were seeded at a density of 1 × 10^5^ cells/mL into a 6-well plate containing a sterile glass cover slip. Following treatment, cells were rinsed with PBS and stained with 2′,7′-dichlorofluorescin diacetate (DCFDA, Sigma-Aldrich) dissolved in 1% FBS in PBS for 30 min at 37 °C in the dark. Post staining, the coverslip containing cells was immediately rinsed twice with PBS, mounted on slides with Fluoromount-G (Invitrogen, Waltham, MA, USA), and imaged using Olympus BX41 microscope (Olympus, Waltham, MA, USA). Three independent biological repeats were conducted. From each biological repeat, three images were taken per treatment, and the fluorescence intensity was quantified using ImageJ software (version 1.53c, National Institutes of Health, Bethesda, MD, USA) using three randomly selected fields per image for each treatment studied. ROS levels were calculated as the relative fluorescence intensity of each treatment against the respective diluent control. 

### 2.9. RNA Isolation and Real Time-PCR (RT-PCR) Analyses

Total RNA was isolated from 3 × 10^5^ HaCaT cells using TRIzol (Invitrogen). cDNA was reverse transcribed from 1 μg RNA using the ThermoScript RT-PCR system (Invitrogen). The RT-PCR primer sequences used (IDT, Chicago, IL, USA) are as follows: catalase (*CAT*): PAO-2027 (forward, 5′-GTGCGGAGATTCAACACTGCCA-3′) and PAO-2028 (reverse, 5′-CGGCAATGTTCTCACACAGACG-3′); superoxide dismutase 1 (*SOD1*): PAO-2025 (forward, 5′-CTCACTCTCAGGAGACCATTGC-3′), and PAO-2026 (reverse, 5′-CCACAAGCCAAACGACTTCCAG); nitric oxide synthase (*NOS2*): PAO-2031 (forward, 5′-GCTCTACACCTCCAATGTGACC-3′), and PAO-2032 (reverse, 5′-CTGCCGAGATTTGAGCCTCATG-3′); and glutathione 3-phosphate peroxidase (*GAPDH*): PAO 230 (forward, 5′-ACTTTGGTATCGTGGAAGGACT-3′), and PAO 231 (reverse, 5′-GTAGAGGCAGGGATGATGTTCT-3′). A 20 µL reaction mixture containing 50 ng of cDNA template, 0.25 μM primers, and 10 μL SYBR Green Master Mix (Applied Biosystems, Carlsbad, CA, USA) was run in QuantStudio 3 Real-Time PCR System (Applied Biosystems) using the following conditions: 95 °C for 10 min followed by 40 cycles of 95 °C for 1 min, 60 °C for 1 min, and 72 °C for 1 min. Fold change in expression was calculated as fold change = 2 − ΔCt_treatment_/2 − ΔCt_vehicle_, where ΔCt = (Ct_target_ − Ct_internal control_). All selected genes were normalized to the expression of internal control, GAPDH. 

### 2.10. Statistical Analyses

All data analyses were performed using the GraphPad Prism software (version 7.05, San Diego, CA, USA). Results were represented as either the mean ± standard deviation (SD) or the mean ± standard error of the mean (SEM). Statistical differences between two or more groups were analyzed using one-way or two-way analysis of variance (ANOVA) followed by Tukey’s or Sidak’s post-test for multiple comparisons, respectively. Statistical significance is stated in the text.

## 3. Results and Discussions

### 3.1. Chemical Analyses of CP Extracts

Previously, we demonstrated the synergistic effect of high pressure and high temperature (HPHT) in increasing dissolution and penetration properties of the solvent, resulting in an enhanced solubility of the target compounds [25,29]. Here, we used an environmentally friendly extraction technique that combines the advantages of HPHT for the extraction of bioactive components from CP. CP were extracted in water (CPW) or water: ethanol (1:1 [*v*/*v*]; CPE) by HPHT for 30 min at 150 °C, as previously described [25]. Currently, there is a growing emphasis on utilizing environmentally friendly extraction methods that align with sustainable practices to achieve maximal recovery of bioactive compounds. Given the safety concerns associated with ethanol-rich solutions, particularly in pilot- and industrial-scale production, and based on our previous studies [27], we opted for one distinct water:ethanol ratio (1:1 [*v*/*v*]) for this study. We evaluated the content of Total Polyphenol (TP), Total Flavonoids (TF), and Antiradical Power (ARP) of tart CP extracts. The results are demonstrated in Table 1. 

The addition of ethanol to the extraction solvent increased the yield of TP by 2.1-fold from 7.20 ± 0.25 mg_CAE_/g_Dried Pit_ in CPW to 15.36 ± 1.88 mg_CAE_/g_Dried Pit_ in CPE. A similar trend was also noted in our prior study, which led to a higher extraction yield of phenolic compounds from different food waste sources, such as olive pomace and grape pomace, using water: ethanol compared to 100% water as extraction solvents [27].

CPE showed enhanced antioxidant power (6.77 ± 1.14 mg_DPPH_/mL_Extract_) when compared with CPW (2.80 ± 0.76 mg_DPPH_/mL_Extract_), as indicated by the ARP assays. The relationship between the total polyphenolic content and the antioxidant activity has been previously reported by our group [29,39] and others [22,40,41]. The results from the ABTS assay also showed a higher antioxidant activity for CPE (1.79 ± 0.00 mg_Trolox_/mL_Extract_) than CPW (0.75 ± 0.04 mg_Trolox_/mL_Extract_). Researchers have reported an antioxidant activity (ABTS) of 2.16 mg_Trolox Equivalent_/g of CP, using water:ethanol 60:40 (*v*/*v*) as the extraction solvent at 70 °C [42]. Few studies have focused on the benefits of bioactive components from different parts of cherries, including the stems [17] and seeds [24]. Analyses of cherry by-products, including cherry pomace and pits, showed high levels of antioxidants, including flavonoids, phenolic acids, and anthocyanins [3]. Another study focused on the composition of CP oil from sour CP [43]. Our findings revealed that for both tart CPE and CPW, TP contents were approximately ten times higher compared to previous studies conducted on tart CP [43]. TF yield increased only by 11.5% from CPW to CPE. Our results showed that the TF content of both CPW and CPE were higher than the TF content reported for five out of six small dark fruits, including bilberries (5.97 mg_Quercetin Equivalent_/g_Dried Biomass_), blueberries (6.28 mg_Quercetin Equivalent_/g_Dried Biomass),_ blackcurrants (8.28 mg_Quercetin Equivalent_/g_Dried Biomass_), redcurrants (4.65 mg_Quercetin Equivalent/gDried Biomass_), cherries (4.76 mg_Quercetin Equivalent_/g_Dried Biomass_), and plums (3.17 mg_Quercetin Equivalent/_g_Dried Biomass_) [30]. In the same study, the highest TP content was measured for blackcurrants (19.84 mg_GAE_/g_Dried Biomass_), followed by cherries (12.92 mg_GAE_/g_Dried Biomass_), bilberries (10.94 mg_GAE_/g_Dried Biomass_), redcurrants (7.38 mg_GAE_/g_Dried Biomass_), blueberries (4.62 mg_GAE_/g_Dried Biomass_), and plums (3.42 mg_GAE_/g_Dried Biomass_) [30]. This comparison highlights the efficiency of our extraction method to recover metabolites such as TP and TF from CP.

### 3.2. Metabolomic Analysis of CP Extracts 

**Figure 1:**
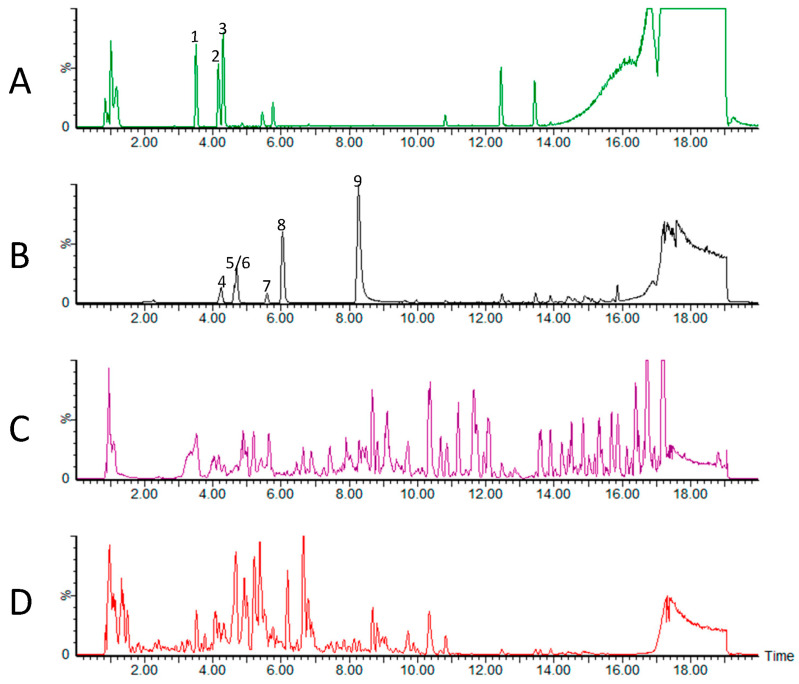
Chromatograms of the standard compounds and CP extracts detected by LC-MS/MS. (**A**) chlorogenic acid isomers (1: trans-5-*O*-caffeoylquinate [neochlorogenic acid], 2: trans-3-*O*-caffeoylquinate, 3: trans-4-*O*-caffeoylquinate [cryptochlorogenic acid]); (**B**) phenolic standards (4: catechin, 5: caffeic acid, 6: amygdalin, 7: *p*-coumaric acid, 8: epicatechin gallate, 9: quercetin); (**C**) CPE: CP extracted in 1:1 [*v*/*v*] water/ethanol; (**D**) CPW: CP extracted in water. Compounds in CPE and CPW were quantified using authentic standards with chromatograms A and B serving as references. The X-axis represents Time (in seconds), while the Y-axis depicts Peak Intensity (%).

To identify the natural compounds present in tart CP extracts, LC-MS/MS metabolomic analyses were performed. Many compounds were detected that could be annotated based on their accurate mass and MS/MS spectra (Figure 1) and confirmed using authentic standards (Table 2). Multiple isomers of caffeoylquinate esters, which include chlorogenic acid, were detected and quantified. In addition, isomers of related compounds were annotated as *p*-coumarylquinate based on accurate mass and fragmentation patterns. Several free hydroxycinnamic acids were also detected and quantified including *p*-coumaric acid, caffeic acid, and ferulic acid. The flavonoids naringenin, quercetin, apigenin, and catechin were also quantified in the CP extracts. In most cases, the levels of compounds were higher in the CPE samples compared to the CPW samples, as the presence of ethanol in the extraction solvent likely facilitated the extraction of the semi-polar phenolic compounds [27]. In addition, the CP extracts also contained a quantifiable amount of the cyanogenic glycoside amygdalin. Likewise, the amygdalin levels were significantly higher (~10-fold) in the CPE as compared to the CPW extracts. Unexpectantly, although the presence of gallic acid was expected in the extracts based on previous studies [31,32,44], the LC-MS/MS analysis did not detect the presence of this compound in both CPE and CPW (Table 2). One potential reason could be linked to the heat sensitivity of gallic acid, leading to its degradation as a result of the extraction temperature [45]. Researchers have shown that gallic acid extraction can initially occur to a certain extent, followed by a decline, as the compound degrades at temperatures exceeding 60 °C, with approximately 30% degradation at 100 °C [46].

Our LC-MS/MS results from tart CP revealed the presence of similar compounds reported in other parts of cherries. Caffeic acid, quercetin, and *p*-coumaric acid were identified in the stems of sweet cherries [31]. Chlorogenic acid, quercetin, and catechin were detected in Ginja cherry stems and leaves [47]. Whole frozen and dried tart cherries of Montmorency and Balaton cultivars have been shown to contain quercetin and catechin [48]. 

Additionally, LC/MS-MS analyses of freeze-dried tart CP extracts used in cellular studies were also analyzed. As expected, metabolites were found in higher concentrations in the freeze-dried samples than in the liquid extracts. Nonetheless, we found that the method of preparation had no significant effect on the qualitative profile of the metabolites (Table 2). Freeze-drying of plant materials is frequently used both in pharmacological and clinical trials to improve the lipophilicity of the extracts [49]. Thus, our findings demonstrate the efficiency of our extraction and preparation methods for further utilization of CP food industry byproducts.

### 3.3. UV–Visible Spectra of CP Extracts

**Figure 2:**
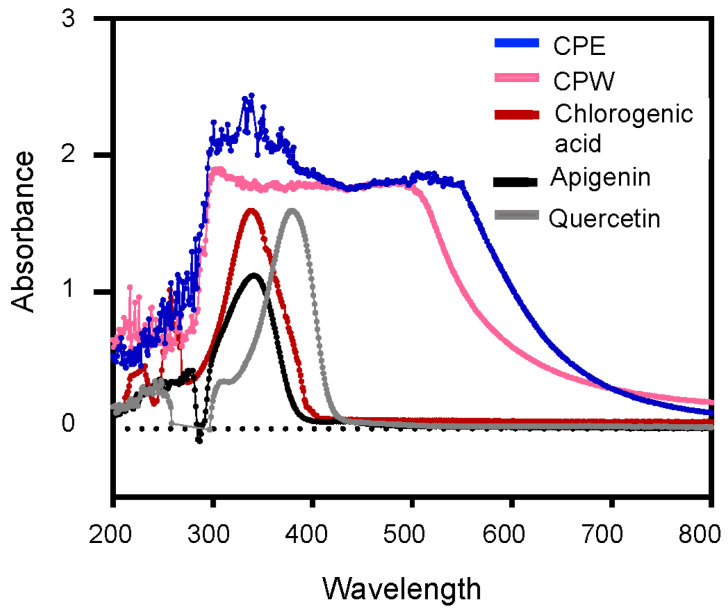
UV–Visible absorption spectra of CP extracts. Absorption spectra of 50 μM CPE or CPW extracts, 80 μM apigenin, quercetin, and chlorogenic acid scanned between wavelengths of 200 and 800 nm. The dotted line at absorbance 0 represents the baseline.

To compare the chemical characteristics of CPE and CPW, freeze-dried extracts were dissolved in DMSO to obtain a concentration of 50 μM equivalent chlorogenic acid and evaluated using UV–Vis spectroscopy between wavelengths of 200 and 800 nm. Pure apigenin, quercetin, and chlorogenic acid were used as standards, each of which showed typical absorption bands within 200–280 nm and a third maximum between 330–440 nm wavelengths (Figure 2), in concordance with previously reported studies [50,51].

CPW and CPE extracts showed similar absorption spectra displaying two main absorption peaks (Figure 2). CPE exhibits a maximum between 300–380 nm with an additional absorption ranging from 450–540 nm, whereas we found that CPW exhibits maxima between 280–330 nm and 440–520 nm. Maxima peaks of CP extracts between 280–380 nm corresponds to the presence of flavonoids, such as apigenin and quercetin, and coumaroyl polyphenols, including chlorogenic acid, evidenced by the profiles of these pure compounds (Figure 2). Together, these results confirm that CP extracts are rich in polyphenols, such as coumaroyl derivatives and flavonoids.

### 3.4. Effect of CP Extracts on Cell Viability

**Figure 3:**
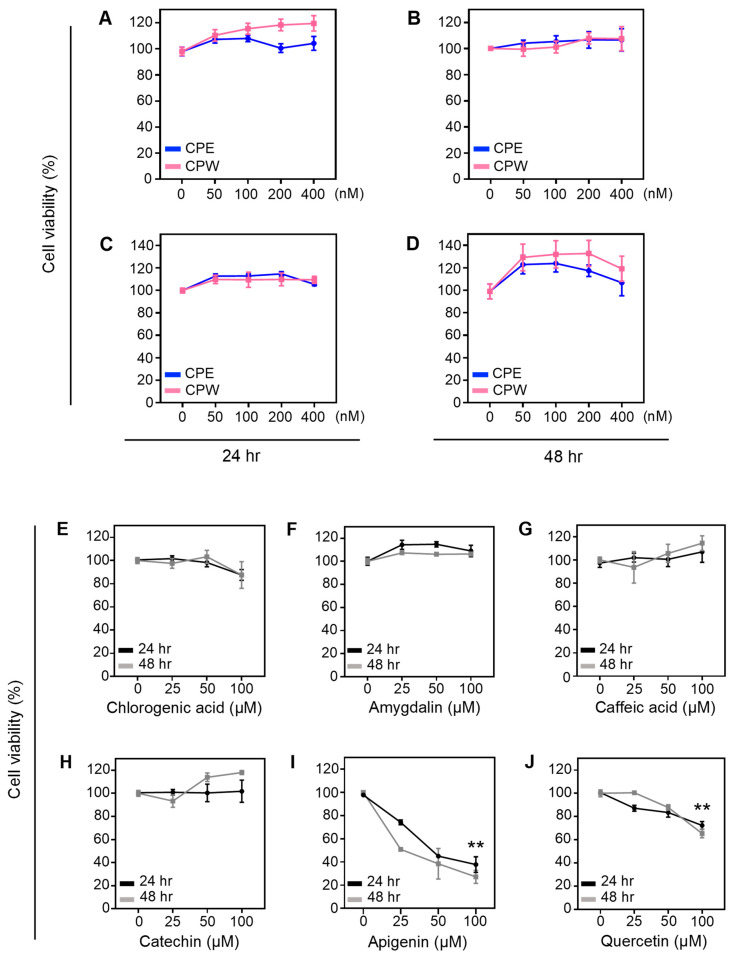
Effect of CP extracts on the viability of human keratinocytes. The percentage of cell viability was determined in human keratinocyte HaCaT cells treated with 50, 100, 200, 400 nM CPE, CPW, or vehicle (DMSO, indicated as 0) using MTS assay for (**A**) 24 or (**B**) 48 h. Cell viability was evaluated in fibroblast NIH3T3 cells treated as above for (**C**) 24 or (**D**) 48 h. Data represent mean ± SEM, *n* = 4–6. The percentage of cell viability was evaluated in HaCaT cells treated with 25, 50, 100 μM (**E**) chlorogenic acid, (**F**) amygdalin, (**G**) caffeic acid, (**H**) catechin, (**I**) apigenin, (**J**) quercetin, or vehicle DMSO (indicated as 0) for 24 or 48 h. Data are represented as mean ± SEM, *n* = 4. ** p < 0.001 compared to vehicle.

To investigate the effect of tart CP extracts on the skin, cell viability was evaluated in human keratinocyte HaCaT cells. We found that increasing concentrations of CPE and CPW ranging from 50, 100, 200, to 400 nM or vehicle (DMSO) did not affect the viability of HaCaT cells after 24 or 48 h (Figure 3A,B, respectively). Similarly, CPE and CPW had no effect on the viability of NIH3T3 fibroblast cells after treatment for 24 or 48 h (Figure 3C,D, respectively). These results indicate that tart CP extracts, at the concentrations tested, have no harmful effects on the viability of skin cells.

To determine if some of the polyphenols found in the tart CP extracts affect skin cell viability, HaCaT cells were treated with pure polyphenols at concentrations commonly used for cell-based assays, ranging from 25 to 100 µM for 24 or 48 h. We found that chlorogenic acid (Figure 3E), amygdalin (Figure 3F), caffeic acid (Figure 3G), and catechin (Figure 3H) did not affect the viability of skin cells as compared to the vehicle (indicated as 0). Apigenin (Figure 3I) and quercetin (Figure 3J) significantly decreased cell viability at 100 µM (*p* < 0.001). Consistent with our results, others have shown that these compounds do not affect the viability of skin cells [52,53,54,55,56,57,58]. Based on our metabolomic results shown in Table 2, the concentrations of these compounds in the highest doses of CP extracts used in the cell viability assays are less than 500 nM, which is at least 50-fold lower than the dosages of the pure polyphenols tested (Figure 3E–J). Therefore, the concentration of abundant compounds found in the dosage of CP extracts does not have deleterious effects on skin cells.

### 3.5. CP Extracts Reduced H_2_O_2_-Induced ROS Levels

**Figure 4:**
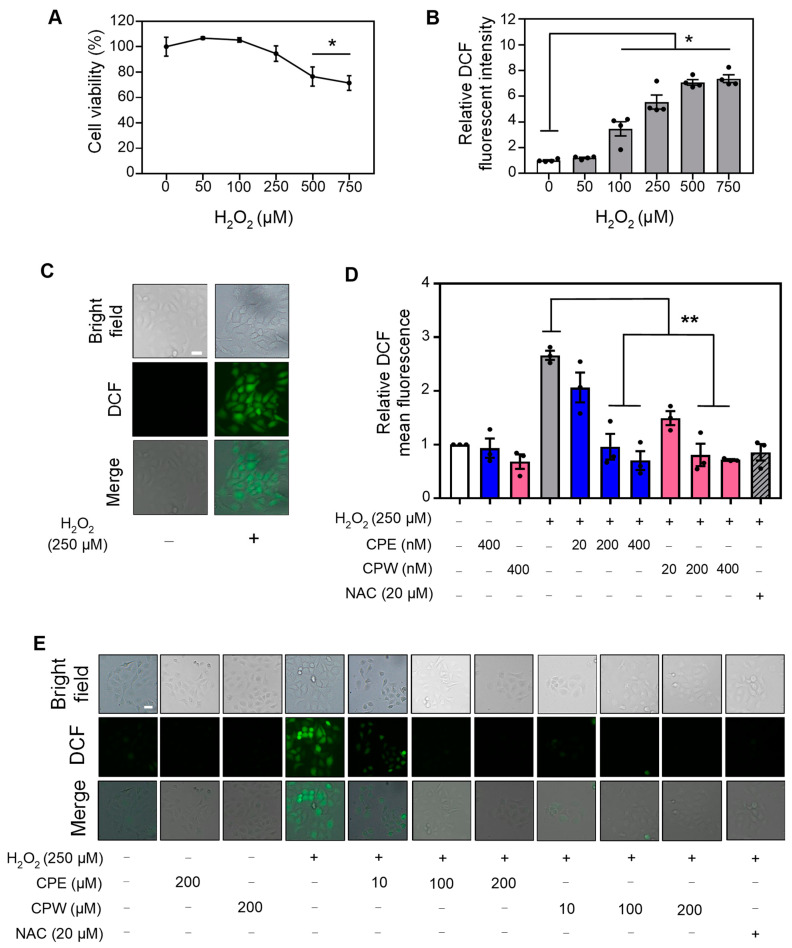
Effect of CP extracts on H_2_O_2_-induced ROS levels in human keratinocytes. HaCaT cells were treated with 50, 100, 250, 500, 750 μM H_2_O_2_ or vehicle (water, indicated as 0) for 24 h. (**A**) Cell viability was calculated using MTS assays. (**B**) Post-treatment, intracellular ROS levels were evaluated using DCF-DA staining, and the relative fluorescence intensity was assessed at excitation and emission of 485 and 520 nm, respectively. (**C**) Representative images depicting ROS levels in HaCaT cells treated with 250 μM H_2_O_2_ or vehicle as in (**B**). (**D**) Relative intracellular ROS levels were evaluated by DCF-DA staining in HaCaT cells pre-treated with 20, 200, 400 nM CPE (blue bars), CPW (pink bars), or vehicle DMSO for 3 h followed by treatment with 250 μM H_2_O_2_ or water for an additional 24 h and visualized by microscopy. Cells pre-treated with the antioxidant NAC (20 μM) were used as a control to evaluate ROS inhibition. (**E**) Representative images depicting ROS levels from data are represented in (**D**). Scale bar: 20 µm. All data depict mean ± SEM, *n* = 3–4. * *p* < 0.05, ** *p* < 0.001 compared to vehicle for (**A**,**B**) and H_2_O_2_ treatment for (**D**).

Previous studies have shown that cherries exhibit antioxidant capabilities [59]. Bioactive components that have been found in cherries, such as chlorogenic acid or apigenin, are known to be ROS-scavengers [52,60]. For example, tart cherry extract was shown to be rich in polyphenols such as chlorogenic acid, quercetin, and kaempferol and suppresses ROS-mediated oxidative stress in HaCaT cells [58]. However, the antioxidant effects of tart CP extracts have not been explored. To study the antioxidant effect of tart CP, we first established the optimal concentration of H_2_O_2_ that is both harmless for skin cell viability and capable of inducing ROS. For this purpose, we first studied the effect of increasing concentrations of H_2_O_2_ from 50, 100, 250, 500, to 750 µM on human HaCaT skin cells for 24 h. We found that the cell viability decreased by approximately 20% at concentrations 500 µM and above (Figure 4A). Next, we evaluated ROS production in HaCaT cells treated with increasing concentrations of H_2_O_2_ for 24 h. We found a significant increase in ROS at 100 µM and higher concentrations of H_2_O_2_, as demonstrated by DCF-DA staining (Figure 4B,C). Based on these results, we selected 250 µM H_2_O_2_ to evaluate the antioxidant effects of CP extracts since this concentration induces significant levels of ROS and does not affect cell viability.

To study if CP extracts have antioxidant activity, HaCaT cells were pre-treated with tart CPE or CPW at concentrations ranging from 20, 200, to 400 nM for 3 h followed by 250 µM H_2_O_2_ for an additional 24 h. The induction of ROS triggered by H_2_O_2_ was inhibited in cells pre-treated for 3 h with NAC, a major free radical scavenger (Figure 4D,E). Our results showed that CPE and CPW significantly reduced ROS levels (Figure 4D,E). Interestingly, CPW at 20 nM seems to have a slightly higher effect on ROS inhibition as compared to CPE, although not statistically significant (Figure 4D,E). Together, these results suggest that CPE and CPW have antioxidant activity reducing H_2_O_2_-induced ROS levels. To date, no studies have reported ROS-inhibiting activity in cherry pits. However, previous observations have confirmed the presence of compounds in cherry seeds that could suppress oxidative stress. For example, Chinese and sweet cherry seeds contain peptides and phenolic compounds with potent antioxidant activity [24,61]. Seeds from other fruits, such as dates and plums, also contain significant levels of phenolics that possess high antioxidant activity [62]. 

### 3.6. CP Extracts Reduced LPS-Induced ROS Levels

**Figure 5:**
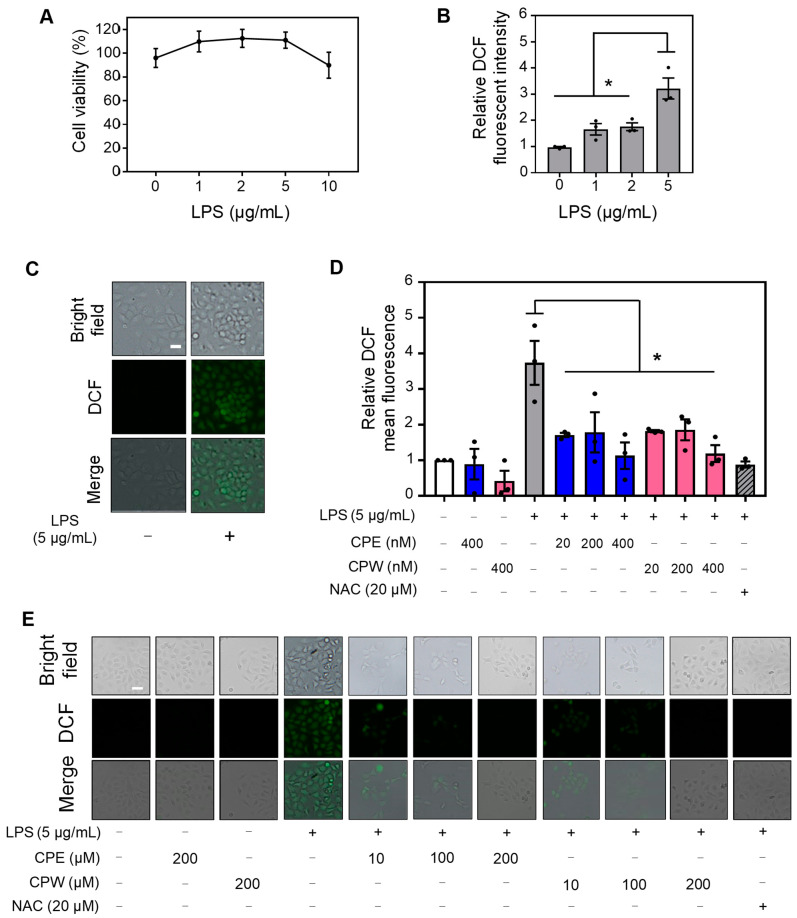
Effect of CP extracts on LPS-induced ROS levels in human keratinocytes. HaCaT cells were treated with 1, 2, 5, 10 μg/mL LPS or vehicle (PBS, indicated as 0) for 24 h. (**A**) Percentage of cell viability was calculated using MTS assays. (**B**) Relative intracellular ROS levels were evaluated by DCF-DA staining. (**C**) Representative images depicting ROS levels from data are represented in (**B**). (**D**) Relative intracellular ROS levels were evaluated by DCF-DA staining in HaCaT cells pre-treated with 20, 200, 400 nM CPE (blue bars), CPW (pink bars), or vehicle DMSO for 3 h followed by treatment with 5 μg/mL LPS or PBS control for an additional 24 h. Cells pre-treated with antioxidant NAC (20 μM) were used as a control to evaluate ROS inhibition. (**E**) Representative images depicting ROS levels from data are represented in (**D**). Scale bar: 20 µm. All data depict mean ± SEM, *n* = 3. * p < 0.05 compared to the vehicle for (**B**) and LPS treatment for (**D**).

Previous studies have shown that tart cherry extracts reduced LPS-induced nitric oxide production [63]. To investigate if CP extracts conferred protection to LPS-induced oxidative stress, we first evaluated its effect on human skin HaCaT viability. To this end, HaCaT cells were first treated with 1, 2, 5, and 10 µg/mL LPS for 24 h. We found that LPS at the concentrations tested had no effect on the viability of the HaCaT cells (Figure 5A). Concomitantly, we evaluated the ROS levels in HaCaT cells when treated with 1, 2, and 5 µg/mL LPS for 24 h by DCF-DA staining. We observed a statistical increase in ROS levels at 5 µg/mL LPS, as indicated by DCF fluorescence (Figure 5B,C). Based on these results, we selected 5 µg/mL LPS as the optimal concentration to evaluate the antioxidant effect of CP extracts against LPS.

To study the impact of CP extracts on LPS-induced ROS levels, HaCaT cells were pre-treated with 20, 200, and 400 nM CPE, CPW, or vehicle (indicated as −) followed by treatment with 5 µg/mL LPS for an additional 24 h. NAC significantly reduced ROS production in LPS-treated HaCaT cells (Figure 5D,E). We showed that pre-treatment with CP extracts decreased ROS levels at all three concentrations tested, as demonstrated by DCF-DA staining (Figure 5D,E). These results suggest that CPE and CPW exhibit antioxidant activity reducing LPS-induced ROS production.

### 3.7. CP Extracts Regulate Antioxidant Gene Expression

**Figure 6:**
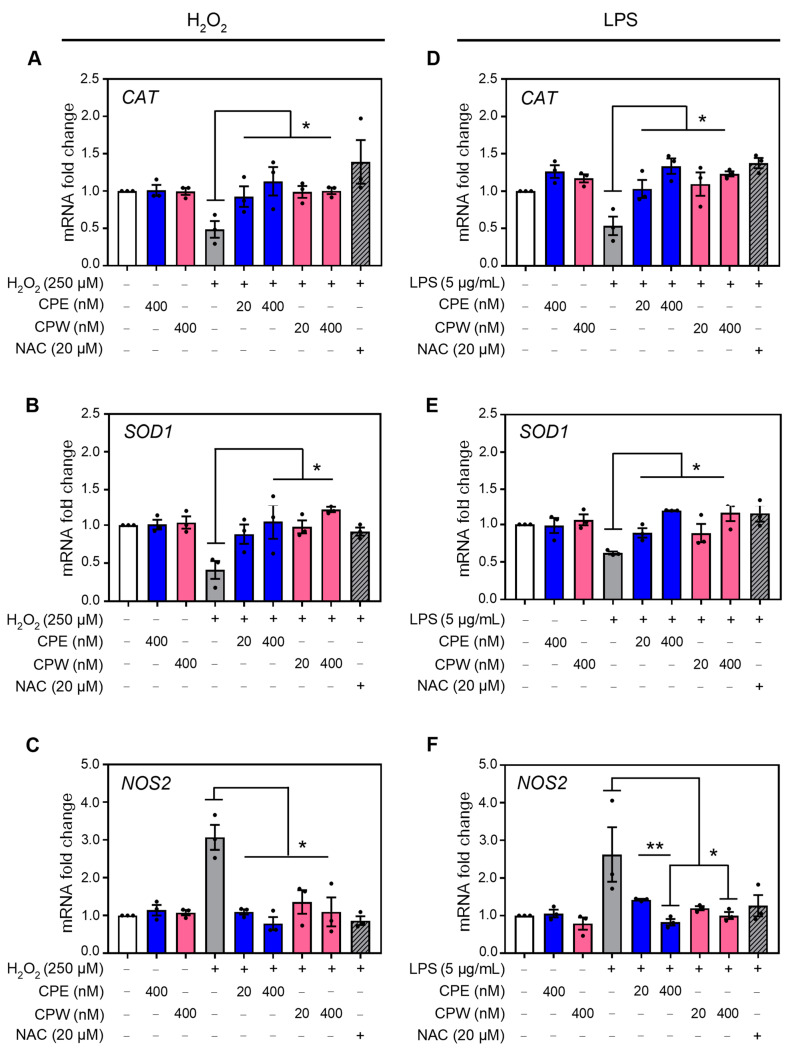
Effect of CP extracts on the expression of ROS-regulating genes in human keratinocytes. Steady-state mRNA levels of *CAT*, *SOD1*, and *NOS2* in HaCaT cells pre-treated with 20 or 400 nM of CPE (blue bars), CPW (pink bars), or vehicle DMSO for 3 h followed by treatment with (**A**–**C**) 250 µM H_2_O_2_, (**D**–**F**) 5 µg/mL LPS, or vehicle for an additional 24 h were evaluated by RT-PCR. Pre-treatment with ROS inhibitor NAC (striped bar) was used as a positive control. Data represent mean ± SEM, *n* = 3. * p < 0.05, ** p < 0.001 compared to H_2_O_2_ or LPS treatment alone (grey bars).

Antioxidant enzymes such as catalase (*CAT*) and superoxide dismutase (*SOD1*) are required to maintain a healthy redox state, which is disrupted during oxidative stress [64,65]. Additionally, an increase in ROS triggers the accumulation of proinflammatory enzyme nitric oxide synthase (*NOS2*) [66]. To investigate the antioxidant mechanisms of CP extracts, the expression of genes encoding these enzymes was evaluated. Treatment with H_2_O_2_ diminished catalase (*CAT*) and superoxide dismutase (*SOD1*) steady-state mRNA levels in HaCaT cells, as previously reported [67]. Pre-treatment with 20 or 400 µM CPE or CPW increased the *CAT* and *SOD1* mRNA steady-state levels compared to H_2_O_2_, as determined by RT-PCR (Figure 6A,B). On the contrary, treatment with either CPE or CPW significantly decreased the steady-state mRNA levels of the pro-oxidant gene *NOS2* by ~1.5-fold compared to cells treated with H_2_O_2_ alone (Figure 6C). As expected, NAC increased *CAT* and *SOD1* and decreased *NOS2* mRNA steady-state levels. Notably, the regulation of *SOD1*, *CAT*, and *NOS2* gene expression by CP extracts was comparable to the effect of NAC, a well-accepted inhibitor or ROS (Figure 6A–C).

Similar results were observed in cells pre-treated with CPE and CPW for 3 h prior to LPS. Treatment with LPS resulted in a decrease in *CAT* and *SOD1* steady-state mRNA expression. Pre-treatment with either CPE or CPW significantly increased *CAT* and *SOD1* steady-state mRNA levels in LPS-treated cells as compared to LPS alone (Figure 6D,E). Both CPE and CPW extracts significantly reduced *NOS2* mRNA levels compared to cells treated with LPS alone (Figure 6F). Importantly, changes in *CAT*, *SOD*1, and *NOS2* steady-state mRNA levels induced by CPE and CPW were comparable to the effect of NAC (Figure 6D–F). Previous studies have reported that chlorogenic acid and flavonoids can enhance the levels of *SOD1* and *CAT* [68,69]. Together these results demonstrate that CPE and CPW extracts counteract the effect of H_2_O_2_ and LPS on the expression of genes responsible for ROS mediation.

## 4. Conclusions

The extraction and utilization of antioxidants from tart CP present a sustainable solution to address the significant waste generated by the food processing industry. With millions of tons of CP being discarded or burned annually, their repurposing not only offers an opportunity to reduce environmental pollution but also provides a sustainable approach aligned with the United Nations 2030 Sustainable Development Goals of responsible consumption and production. Through comprehensive analyses, this study revealed the potential application of antioxidants extracted from CP, a byproduct of the cherry industry, particularly in natural-based skin care products. Metabolomic analyses of the CP revealed a rich profile of antioxidants, including flavonoids, phenolic acids, and anthocyanins, which are known for their anti-inflammatory, ROS-scavenging properties, and other health benefits. Cell-based studies using human keratinocyte cells showed the ability of CP extracts to reduce ROS levels induced by different stimulants, indicating their potential to mitigate inflammation and protect against oxidative stress-induced damage. Although the industrial preparation conditions of the CP used in this study, including the environmental factors, filtration, clarification, etc., have not been considered, the results of this study highlight that by adopting extraction techniques that maximize the recovery of bioactive compounds from CP, manufacturers can reduce waste and minimize the environmental impact of their operations while providing novel and natural antioxidants products for the regulation of skin homeostasis.

## 5. Future Recommendations

It is worth noting that the composition of polyphenolic-enriched final cosmetic products can vary significantly depending on the specific formulation used. While the formulation of cream was not within the scope of our current research, we acknowledge that further exploration and characterization of extracts, product formulation, product efficacy, and a comprehensive life cycle assessment analysis considering the mass and energy consumption are warranted to fully realize the opportunities of CP extracts for a sustainable cosmetic industry.

## Figures and Tables

**Table 1:** UV–Visible spectroscopy analysis of tart CP extracted in water (CPW) and 1:1 [*v*/*v*] water: ethanol (CPE). Total Polyphenols (TP), Total Flavonoids (TF), and Antioxidant Activity (DPPH and ABTS) were measured using colorimentric assays.

Assay	CPW	CPE
TP Yield (mg_Caffeic Acid Equivalent_/g_Dried Pit_)	7.20 ± 0.25	15.36 ± 1.88
TF Yield (mg_Quercetin Equivalent_/g_Dried Pit_)	6.95 ± 0.81	7.75 ± 1.11
DPPH (mg_DPPH_/mL_Extract_)	2.80 ± 0.76	6.77 ± 1.14
ABTS (mg_Trolox Equivalent_/mL_Extract_)	0.75 ± 0.04	1.79 ± 0.00

*n* = 3. Data represent mean values ± SD. DPPH: 2,2-diphenyl-1-picryl-hydrazyl-hydrate. ABTS: 2,2′-azinobis-(3-ethylbenzothiazoline-6-sulphonic acid).

**Table 2:** Metabolomic analyses of tart CP extracts. Chemical compounds found in CP extracts and freeze-dried CP extracts. CPW: CP extracted in water. CPE: CP extracted in 1:1 [*v*/*v*] water/ethanol.

Compound Name	Concentration in CPW (μg/mL)	Concentration in CPE (μg/mL)	Concentration in Freeze-Dried CPW in DMSO (μg/mL)	Concentration in Freeze-Dried CPE in DMSO (μg/mL)
*p*-Coumarylquinate isomer 2	10.42	13.84	50.08	80.38
*p*-Coumarylquinate isomer 3	0.95	0.85		
*p*-Coumarylquinate isomer 1	4.43	5.45	23.67	36.34
Chlorogenic acid	4.89	17.43	18.07	11.52
Cryptochlorogenic acid	3.76	0.96	17.35	31.97
Neochlorogenic acid	2.69	0		
*p*-Coumaric acid	0.87	2.69	1.69	6.7
Amygdalin	0.58	4.25	1.26	7.72
Ferulic acid	0.44	1.35	0.17	0.56
Caffeic acid	0.15	0.47	0.24	1.04
Apigenin	0.01	0.69	0.27	20.27
Naringenin	0.01	0.34	0.65	31.20
Quercetin	0	0.06	0.18	0.29
Catechin	0	2.54	0	2.77

## Data Availability

The authors confirm that the data supporting the findings of this study are available within the article. All other relevant data supporting the findings of this study are available from the corresponding authors upon reasonable request.
